# Exposure to maternal nicotine *in utero* and/or via lactation alters craniofacial development in mice

**DOI:** 10.1371/journal.pone.0329403

**Published:** 2025-08-01

**Authors:** Amr Mohi, Emily L. Durham, Rajiv Kishinchand, James J. Cray Jr

**Affiliations:** 1 Department of Cell Systems and Anatomy, University of Texas Health San Antonio Long School of Medicine, San Antonio, Texas, United States of America; 2 Division of Human Genetics, Department of Pediatrics, Children’s Hospital of Philadelphia, Philadelphia, Pennsylvania, United States of America; 3 Divisions of Biosciences and Orthodontics, The Ohio State University College of Dentistry, Columbus, Ohio, United States of America; 4 Department of Biomedical Education and Anatomy, The Ohio State University College of Medicine, Columbus, Ohio, United States of America; Bangladesh Agricultural University, BANGLADESH

## Abstract

The Center for Disease Control’s National Birth Defects Prevention Study data suggests that maternal nicotine use may increase the incidence of craniofacial birth defects and growth anomalies like craniosynostosis, cleft palate, and/or lip in offspring. Craniofacial growth proceeds by expansion at fibrous sutures and synchondroses. In the cranial base, synchondroses, which are cartilaginous joints, play a major role in craniofacial development including neurocranial expansion and facial outgrowth. Our previous data showed alterations in craniofacial structures with intrauterine exposure to nicotine. As the use of nicotine is increasing among youths, especially through use of electronic nicotine delivery systems, there is a great need to investigate the critical periods of nicotine exposure during pregnancy and postnatal development. Alterations in craniofacial growth that occur in response to maternal nicotine use must be understood in order to prevent debilitating conditions. For this investigation, we utilized cephalometric and histomorphometric analyses to investigate how nicotine exposure alters craniofacial development in offspring modeling maternal nicotine exposure either during pregnancy and lactation or post-partum lactation only compared with unexposed controls. Our results in mice showed significant changes in craniofacial dimensions and some specificity for effects in the synchondroses across the three experimental groups including significant changes in the cellular and the extracellular collagen matrix components of these growth centers. The most dramatic effects segregated to the lactation only exposed group, which is a major target from a prevention point of view as there is a common misconception among the public that nicotine cessation during pregnancy is sufficient for prevention of ill effects in the offspring.

## Introduction

According to the United States Centers for Disease Control, 1 child in every 33 is born with a birth anomaly [1]. Birth anomalies account for 20% of all infant deaths making them the leading cause of infant deaths in the United States [1]. Craniofacial anomalies which are congenital differences affecting cranial and/or facial structures account for a major class of birth anomalies. These debilitating conditions range from mild to severe and can cause a wide range of facial dysmorphology, feeding disorders, airway obstruction, and potentially present life-threatening neurological complications. Craniofacial birth anomalies include various cleft lip and/or palate phenotypes, craniosynostosis, facial malformation, and various other defects [1–6]. These craniofacial conditions can be part of a syndrome (syndromic) due to a genetic mutation or non-syndromic because of an environmental exposure to a teratogen or the result of gene-environment interactions [7].

Previous studies have shown that craniofacial tissue disruption and non-syndromic abnormalities can occur as a result of exposure to teratogens such as tobacco smoking or nicotine exposure [8–13]. For decades tobacco smoking has been the target of nationwide efforts to spread awareness of its adverse health effects in efforts to reduce morbidity and mortality [11]. It is well established that embryonic nicotine exposure adversely affects development potentially through inhibition of specific potassium channels [10,14–21]. Nicotine can pass through the placenta and/or through breastmilk and cotinine levels in fetus and baby can be higher than those in the mother, particularly with high nicotine use [18,22–25]. Public Health efforts are now facing an impact due to the rise in electronic nicotine delivery system (ENDS) use, including E-Cigarettes and other vaping devices. Recent studies have highlighted a concern that most users of ENDS are young including individuals of childbearing age and younger [12,26,27]. It is likely that high use of ENDS in this young group is driven by flavorings and targeted marketing. Importantly such additives may also have detrimental effects [18,28,29]. Though there is a perception that these ENDS are safer than tobacco smoking, they contain higher concentrations of nicotine than traditional cigarettes and other tobacco products [30]. Further, the prevalence of beginning nicotine use during lactation after abstaining during gestation due to perceived safety is poorly understood but is greater than 10% of child-bearing individuals [23]. With ENDS now proliferating the market, and tobacco companies having consistent sales in use of traditional nicotine delivery products, there is a great need to study the effect of nicotine, the common ingredient in traditional and emerging products.

In this study we used a nicotine exposure murine model that mimicked active nicotine use during pregnancy and/or breast feeding. Use of nicotine replacement therapy products during pregnancy is common as they are advertised as a safe alternative to cigarette smoking [23,31–34]. In our experiments we investigated a control group of unexposed mouse pups, and two groups of pups exposed to nicotine. One group was exposed to nicotine during pregnancy and lactation, and the other group was exposed during lactation only to mimic the most common patterns of nicotine use coinciding with pregnancy. As the direct impact of nicotine on craniofacial development has yet to be understood beyond the presence of anomalies and altered cell function [17,19–21,25,35–37], we investigated cranial growth sites (sutures) and centers (synchondroses) for aberrant changes in morphology and any osseous obliteration or fusion that would negatively affect the trajectories of craniofacial growth. Our cephalometric and histologic analyses sought to test if nicotine exposure can alter craniofacial growth specifically at sutures and synchondroses [38–39]. Our hypothesis was that nicotine when introduced *in utero* or during lactation would disrupt craniofacial growth, with pregnancy and lactation exposures logically exhibiting the greatest effects due to the prolonged exposure.

## Materials and methods

### Mouse model and nicotine exposure

Adult wild type, C57BL6J (Mus musculus, Jackson Laboratories, Bar Harbor, ME) male and female mice were utilized to produce litters. Control mice (n = 24) were produced from breeding pairs that were not exposed to nicotine. To mimic the effects of recurrent nicotine exposure to the fetus and/or pups as in maternal smoking and other nicotine related exposures, nicotine (Sigma Aldrich N3876, St. Louis, MO, N3876) was diluted in drinking water at 100 μg/ml throughout the proposed exposure times (pregnancy and lactation (n = 25) or only postnatally throughout lactation (n = 26)) (Table 1) [10,13,15,40]. This dose was chosen based on previous research by our laboratory as one in which cotinine levels were representative of an active nicotine user [10,15,41–45]. All animals were monitored daily by the research team and animal laboratory staff for signs of dehydration and distress. At least 3 litters from each exposure group were used for these analyses to reduce any effect of the litter. Pups from all three groups under study (n = 75) were euthanized at postnatal day 15 via carbon dioxide inhalation and subsequent decapitation in accordance with National Institutes of Health guidelines to alleviate animal subject suffering. Blood serum to assess nicotine consumption was isolated according to manufacturer protocol (Greiner Bio-one, Kremsunster, Austria, MiniCollect Z Serum Sep.) from dams and pups at euthanasia. Blood collection, and assessment of nicotine metabolism via cotinine ELISA was performed according to manufacturer protocol (Calbiotech, El Cajon, CA, USA, CO096D-100) [15,46]. Skulls were fixed with 4% paraformaldehyde for 48 hours, then switched to 70% Ethanol for micro-computed tomography (μCT) and finally processed for paraffin-based histology. Animal use protocols were approved by the Medical University of South Carolina Institutional Animal Care and Use Committee (AR#3510). All breeding procedures were carried out in an Association for Assessment and Accreditation of Laboratory Animal Care International accredited facility where all husbandry and related services were provided by the Division of Laboratory Animal Resources. All procedures follow the guidelines of Animal Research Reporting In vivo Experiments “ARRIVE” meet all applicable standards for the ethics of experimentation and research integrity [47].

**Table 1:** Sample Details.

Exposure	Number of Litters included (pups/litter)	n	Sex	Weight (g)	Cotinine^1^ Mean + /-SE
Control	7 (1-6)	24	Male – 11 Female – 13	Male – 7.2385 +/- 0.1922 Female – 6.801 + /- 0.2243 Average – 7.0015 +/- 0.1538	
Preg + Lac	4 (5-9)	25	Male – 13 Female – 12	Male – 7.0000 +/- 0.1961 Female – 7.0000 + /- 0.2132 Average – 7.000 +/- 0.1386	11.5900 +/- 1.1600
Lac Only	3 (8-9)	26	Male – 13 Female – 13	Male – 6.1538 +/- 0.1538 Female – 6.5384 + /- 0.2432 Average – 6.3462 +/- 0.1433	7.1000 +/- 1.8700^**2**^

^**1**^ Cotinine level in pregnant dams was 134.3816ng/ml + /- 3.81. Active smocking is replicated with serum cotinine levels above 10ng/ml.

^**2**^ Pups in the lactation only exposure litters were significantly smaller than other exposures (p = 0.002).

### Micro-computed tomography (μCT)

As previously described [48,49] μCT images were obtained on postnatal day 15 mouse skulls with a Sky Scan 1174 (Kontich, Belgium) at a 22.57 μm voxel resolution. Mouse skulls were reconstructed with CT Vox software v2.3.0 r810 (Sky Scan). Analyze-Pro software was used for visualizing and measuring areas of interest. Threshold settings were set to only visualize bone volume within the skull. Our categories of measurements obtained from the μCT scans are general cephalometric measures (Table 2), cranial suture widths, and dimensions of the cranial base (synchondroses). The width of the coronal, sagittal, and posterior interfrontal sutures were measured at 25, 50, and 75% of their length then averaged to a single representative width. Three dimensions of the Spheno-occipital synchondrosis and intersphenoidal synchondrosis were measured as following: right to left dimension (width); height at the midline; and the antero-posterior dimension “Patency” at 25, 50, and 75% of its length then averaged to a single representative measure. The described measurements were obtained using the Analyze-Pro software. Presented figures were made with 3D Slicer version 5.2.2.

**Table 2:** Cephalometric Measures.

Measure	Description
Pa – Na	Cranial length – Parietal point to Nasion
Fp – Os	Neurocranium height – Fronto-parietal suture to Occiput
X – Ps	Cranial base length – Anterior sphenoid cranial base bone to posterior occipital cranial base bone
Cw – Cw	Maximum cranial width – Bilateral distance between most lateral points of cranial vault
Zp – Zp	Viscerocranium [facial] width of the zygomatic arch at the posterior end – Bilateral distance between posterior junction of the zygomatic arch & temporal bone
Mp – Mp	Viscerocranium width of the zygomatic arch at the midpoint – Bilateral distance between the anterior-posterior midpoints of the zygomatic arches
Ms – Ms	Viscerocranium width of the zygomatic arch at the anterior end – Bilateral distance between the anterior junction of the zygomatic arch & maxillary process
Op – Rh	Craniofacial length – Opisthion [posterior rim of the foramen magnum] to the most anterior point of the mid palatal suture

### Hematoxylin and eosin synchondroses cell count

Representative samples from each experimental group were decalcified in 0.25 M EDTA at pH 7.4 for 9 days. Samples were placed in fresh EDTA every 3 days (3 total changes). Samples were then washed, dehydrated in graded ethanol (70−100%), cleared in xylene, and embedded in paraffin. Prior to embedding, samples were bisected at the midsagittal plane and placed in the paraffin wax block with the medial sides on the surface to be cut. Eight μm sections were cut using a rotary microtome prior to mounting on Super Frost Plus (ThermoFisher Scientific, Watham, MA, USA) glass slides. Harris’ hematoxylin (VWR, Radnor, PA, 10143−606) and Eosin (VWR, 95057−848) staining proceeded by our standard protocol (10). Representative sections from specimens of the control group (n = 7), the lactation only exposed group (n = 5), and the pregnancy and lactation exposed group (n = 5) were used for histomorphometric analysis to visualize and count chondrocytes of the two cranial base synchondroses: Spheno-occipital synchondrosis (SOS) and intersphenoidal synchondrosis (ISS). Stained sections were photographed using Olympus IX73 compound microscope in brightfield using Cell Sens software. Image J Software (National Institutes of Health) [50] was used to measure the surface area of synchondroses. Cell count was done manually using a stylus on a touch screen Android-13 device to mark and count lacunae/chondrocytes from the captured images inside the area measured in Image J. The number of cells per surface area (millimeter square) was used for data analysis.

### Picro Sirius Red – Synchondrosis Collagen Quantification

To assess collagen amount and maturity, Picro-Sirius red staining was performed per standard methodology as previously described [51]. Stained sections were photographed in brightfield and using polarized light to assess collagen maturity with an Olympus IX73 compound microscope and attached DP74 color CMOS Camera integrated into the capture workstation via Olympus Cell Sens software. Quantities of green (immature), yellow (intermediate), and red (mature) collagen fibers were quantified in the Spheno-occipital and the intersphenoidal synchondroses.

### Statistical analysis

Statistical analysis was conducted using SPSS 28.0 (IBM, Armonk, NY, USA). Data were screened for normality and homogeneity of variance. If these assumptions were met, analyses followed using a one-way ANOVA with post hoc Bonferroni analyses. If assumptions were violated a non-parametric approach was utilized with Kruskal Wallis and post hoc Bonferroni analyses. Differences were considered significant if p ≤ 0.05. All measurements are presented as mean ± SEM.

## Results

Our sample was balanced between male and female mouse pups for each exposure (Table 1) and an analysis of serum cotinine, the major metabolite of nicotine, indicated that these exposures for the most part mimicked active smoking (cotinine in serum >10ng/ml). The lactation only group presented with lower cotinine levels that were still reliably measured in the pups. Pups in the lactation only group were also smaller (p = 0.002) than their pregnancy and lactation exposed (p = 0.006) and control (p = 0.007) counterparts perhaps reflecting larger litter sizes for the lactation only group, or some effect on feeding caused by the nicotine exposure [15,23,44]. No statistically significant differences in size or form of pups were identified by sex (S1 Table). Consideration of litter as a covariate was also interrogated and is included in Supplement (S2 Table).

**Fig 1:**
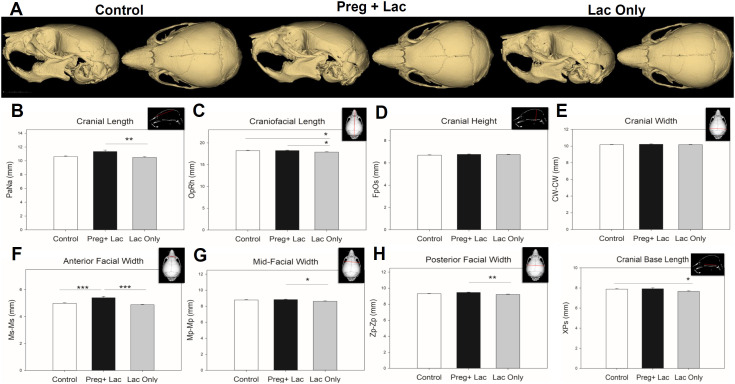
Effects of nicotine exposure during gestation and lactation or only lactation on developing murine craniofacial form. Representative 3D reconstructions from each exposure group highlight the subtle changes due to different nicotine exposures. Left lateral (left) and superior (right) images are presented from left to right for control, Peg + Lac, and Lac Only exposures. Scale = 9 mm (A). Cephalometric analysis showing significant statistical differences between groups are indicated by a black line and asterisk(s). Depictions of each measure are shown inset to the upper right of each plot. Cranial length (B) and craniofacial length (C) indicate a potential lag in growth due to nicotine exposure postnatally (Lac Only, Grey bars). No significant differences were detected in the calvarial height or width (D-E). The lactation only nicotine exposure resulted in a significant reduction in facial width when compared to the pregnancy and lactation exposure (Preg+Lac, Black bars, F-H). The pregnancy and lactation nicotine exposure resulted in a significant increase in anterior facial width (F) when compared to the unexposed control and lactation only groups (Preg+Lac, Black bars). The cranial base length was also significantly shorter in the lactation only exposed pups as compared to control pups (I). n = 24 Control, 25 Preg + Lac, 26 Lac Only *p ≤ 0.05 **p ≤ 0.01 ***p ≤ 0.001.

Our cephalometric analyses (Fig 1) compared skull form in postnatal day 15 control (no exposure), pregnancy and lactation exposed, and lactation only exposed mice and demonstrated statistically significant changes in most of the craniofacial dimensions. Mean cranial length was significantly different (p = 0.012) across the three groups. Bonferroni post-hoc analysis determined that the mean length of the neurocranium of the lactation exposed pups was significantly (p = 0.01) shorter than the individuals exposed during pregnancy and lactation (Fig 1B). Mean craniofacial length was significantly different (p = 0.008) across the three groups. Bonferroni post-hoc analysis determined that the mean full skull length (Op-Rh) of the lactation exposed pups was significantly shorter than both the individuals exposed during pregnancy and lactation (p = 0.016) and the controls (p = 0.026) (Fig 1C). There were no significant differences in cranial height (Fp-Os) (p = 0.340) or cranial width (Cw-Cw) (p = 0.502) across or between our three exposure groups (Fig 1D, E). All facial width measurements showed significant differences between groups (Fig 1F–H). The anterior facial widths where the zygomatic arch ends at the maxilla (Ms-Ms) were significantly (p < 0.001) different across the three groups. Bonferroni post-hoc analysis determined that the anterior facial width (Ms-Ms) in the pregnancy and lactation exposed pups was significantly (p < 0.001) wider than the control pups and the pregnancy and lactation exposed pups (p < 0.001). Likewise, the mean mid-facial width (Mp-Mp) was significantly (p = 0.012) different across the three groups. Bonferroni post-hoc analysis determined that the mean mid-facial width of the lactation exposed pups was significantly (p = 0.013) narrower than the pregnancy and lactation pups. Additionally, the mean posterior facial width (Zp-Zp) was significantly different (p = 0.002) across the three groups. Bonferroni post-hoc analysis determined that the mean posterior facial width (Zp-Zp) in the lactation exposed pups was significantly (p = 0.002) narrower than the pregnancy and lactation exposed individuals. Our data also showed that the mean length of the cranial base (X-Ps) was significantly (p = 0.026) different across the three groups. Bonferroni post-hoc analysis determined that the mean length of the cranial base (X-Ps) in the lactation exposed pups was significantly (p = 0.044) shorter than the controls (Fig 1I).

**Fig 2:**
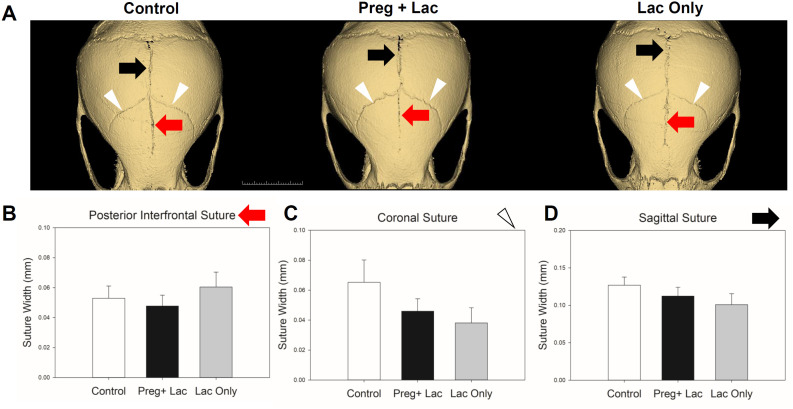
Effects of nicotine exposure during gestation and lactation or only lactation on cranial suture patency. 3D renderings of representative mouse sutures from each exposure group. White arrowheads indicate coronal sutures, black right facing arrow identifies sagittal suture, and red left facing arrow indicates posterior interfrontal suture (A). Mean width (patency) of posterior interfrontal suture (B), sagittal suture (C), and coronal suture (D) indicates a high degree of variability and no statistically significant differences across or between groups. n = 24 Control (White bars), 25 Preg + Lac (Black bars), 26 Lac Only (Grey bars).

**Fig 3:**
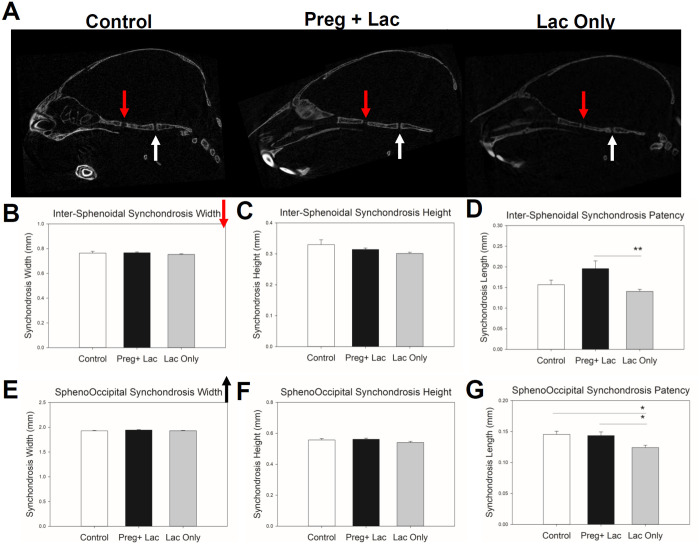
Effects of nicotine exposure during gestation and lactation or only lactation on cranial synchondrosis form. Micro-CT midline sections display ISS (red downfacing arrow) and SOS (white upfacing arrow) in three exposure groups. Analysis did not show any changes in the width or height of the ISS (B-C) or the SOS (E-F). The antero-posterior (patency) showed significant difference across the 3 groups in the SOS (G) and between the pregnancy and lactation and lactation only exposures in the ISS (D). n = 24 Control (White bars), 25 Preg + Lac (Black bars), 26 Lac Only (Grey bars) *p ≤ 0.05 **p ≤ 0.01.

Comparison of changes to the cranial growth sites (sutures) of the skulls of postnatal day 15 control (no exposure), pregnancy and lactation exposed, and lactation only exposed mice and (Fig 2) did not demonstrate any significant differences across or between exposure groups. Further, our data did not show any significant change in the mean height and width of both studied synchondroses but showed significant changes in their patency (Fig 3). Mean patency of the ISS was significantly (p = 0.007) different across the three groups. Bonferroni post-hoc analysis determined that the mean patency of ISS in the lactation exposed pups was significantly (p = 0.006) reduced compared to the pregnancy and lactation exposed pups (Fig 3D). Mean patency of SOS was significantly (p = 0.005) different across the three groups. Bonferroni post-hoc analysis determined that the mean patency of SOS in the lactation exposed pups was significantly reduced compared to both the control (p = 0.011) and the pregnancy and lactation exposed pups (p = 0.019) (Fig 3G).

**Fig 4:**
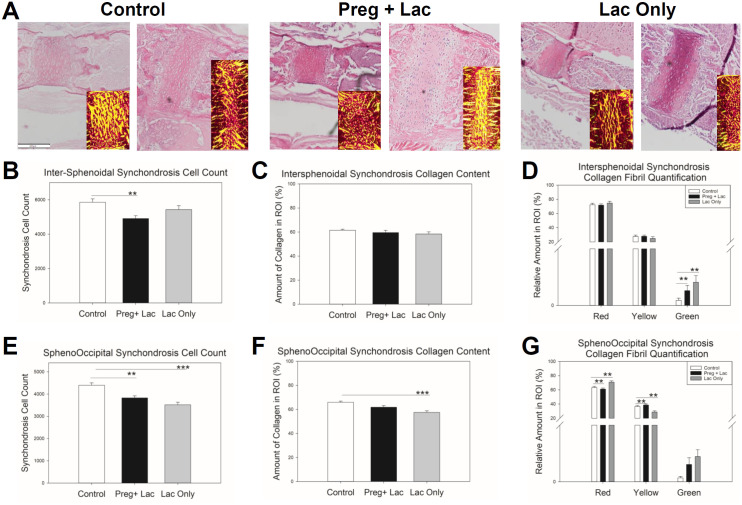
Effects of nicotine exposure during gestation and lactation or only lactation on cellular content of the cranial synchondroses. Representative H&E-stained ISS (left) and SOS (right) with polarized PSR stained images inset (Scale Bar = 200 µm) (A). The analysis of chondrocyte counts per square mm in the ISS showed a significant reduction in chondrocytes in the pups exposed to nicotine during pregnancy and lactation (B). No significant differences were noted in the ISS for total collagen content, however more immature fibers were observed in nicotine exposed pups (C-D). Significantly reduced chondrocyte counts were found in the SOS of pups exposed to nicotine (E). Picro Sirius Red stained section analysis indicated reduced collagen content in the lactation only exposed group SOS. Our analysis also showed a significant reduction in intermediate collagen but a significant increase in mature collagen in the lactation exposed group when compared to the other 2 groups (G). n = 7 Control (White bars), 5 Preg + Lac (Black Bars), 5 Lac Only (Grey Bars) *p ≤ 0.05 **p ≤ 0.01 ***p ≤ 0.001.

Analyses of the cellular (chondrocyte) content of synchondroses in postnatal day 15 control (no exposure), pregnancy and lactation exposed, and lactation only exposed mice was done using the number of cells per area (millimeter square) of the H&E-stained tissue sections. Mean chondrocyte count of the intersphenoidal synchondrosis (ISS) was significantly (p = 0.006) different across the three groups. Bonferroni post-hoc analysis determined that the mean chondrocyte count of ISS in the pregnancy and lactation exposed pups was significantly (p = 0.005) reduced compared to the controls (Fig 4B). Mean chondrocyte count of the of the SOS was significantly (p < 0.001) different across the three groups. Bonferroni post-hoc analysis determined that the mean chondrocyte count of SOS in the lactation only exposed pups was significantly (p < 0.001) reduced compared to the controls, and that the chondrocyte count of SOS in the pregnancy and lactation exposed pups was significantly (p = 0.001) reduced compared to the controls (Fig 4E).

Picro Sirius Red (PSR) stained tissue sections allowed for quantification of collagen content of the ISS in postnatal day 15 control (no exposure), pregnancy and lactation exposed, and lactation only exposed mice. The mean collagen content percentage showed no differences across the three groups (p = 0.369) (Fig 4C). When we quantified the fractions of mature (red), intermediate (yellow), and immature collagen in the ISS, our data did not show any significant differences in the amount of mature (p = 0.615) and the intermediate (p = 0.558) collagen across the three groups, but a significant (p = 0.007) difference in immature collagen content across the three groups was identified. Bonferroni post-hoc analysis demonstrated that the immature collagen of ISS in the nicotine exposed pups was significantly (p = 0.011) higher than the control pups (Fig 4D). Quantification of collagen content of the SOS was significantly (p < 0.001) different across the three groups and the mean collagen content of SOS in the lactation only nicotine exposed pups was significantly reduced compared to the controls (p < 0.001) (Fig 4F). When we quantified the fractions of mature (red), intermediate (yellow), and immature collagen in the SOS, our data showed that the mean percentage of mature collagen in SOS is significantly (p < 0.001) different across the three groups and that the mean percentage of mature collagen in the lactation only exposed pups was significantly (p = 0.006) higher than the controls. Mature collagen was decreased in pups with the longer-term exposure (pregnancy + lactation) compared to control (p = 0.001). Mean intermediate collagen was significantly (p < 0.001) different across the three groups and the mean intermediate collagen of the SOS in the lactation exposed pups was significantly (p = 0.003) less than the controls. In contrast to mature collagen, mean intermediate collagen in the pregnancy and lactation exposed pups was significantly greater (p = 0.001) as compared to control. Mean immature collagen content of the SOS was significantly (p = 0.031) different across the three groups but did not show any significant changes in the immature collagen in SOS between exposures (Fig 4G).

## Discussion and conclusion

We detected significant but not graded changes with the exposure to maternal nicotine only during lactation and during pregnancy and lactation. Interestingly, in some comparisons, only the lactation exposure led to measured statistically significant changes. This aligns with inconsistent results of nicotine exposure in the literature potentially indicating that the effects of nicotine are multi-factorial and highlighting the importance of this work from a public health perspective [10,15,18,19,35,37]. The cranial height and width were not altered by nicotine exposures, but the lactation exposure led to a shorter neurocranium compared to the pregnancy and lactation exposure, while none of the nicotine exposures were shorter than the unexposed controls. Our data demonstrated that in general the neurocranium which is the part of the skull surrounding the brain was not altered by the nicotine exposures at the age investigated here. This was also reflected in our analysis of the distance between bony ends (patency) of the posterior interfrontal, coronal, and sagittal sutures that did not show any fusion (osseous obliteration), significant narrowing, or increase in area. These results are similar to a previous study published by our lab, where exposure to the same concentration of nicotine during pregnancy did not alter cranial height or cranial index (measure of space of neurocranium) of postnatal day 15 mice [10,13]. Again, these mixed results may highlight potential for compensatory growth as a rescue for teratogenic insults during complex craniofacial and neurocranial development. Likewise, these results may indicate that cells other than those needed for neurocranial expansion/ development are affected by nicotine exposure [17,18,35–37,52]. Overall, these results indicate that any exposure to nicotine is potentially hazardous and more specifically resuming nicotine consumption immediately following pregnancy may have adverse effects on offspring.

By focusing on facial length with reference to the neurocranium and the full craniofacial length, our data showed that the anteroposterior dimension of the face (length) is significantly affected by the exposure to maternal nicotine during the lactation period. We also detected significant narrowing of posterior, mid, and anterior face with the lactation exposure. Interestingly this phenotype is consistently found in syndromes or anomalies associated with hypoplastic (shorter) midface which is generally caused by abnormalities in the cranial base and/or prematurely fused synchondroses [39,53]. Our µCT analysis showed that the cranial base length is significantly reduced with the lactation exposure to nicotine compared to controls. In addition to the overall shorter cranial base, the lactation exposure to nicotine caused significant decreases in the anteroposterior dimensions of the ISS and SOS. Synchondroses are growth centers capable of driving craniofacial growth especially in the anteroposterior direction but also height and width [54]. Our results support this theory hence the significant reduction in the anteroposterior craniofacial dimension is matched by an anteroposterior reduction in cranial base and synchondroses dimensions. This is similar to other research indicating that exposure to nicotine containing products correlates with a decrease in bone length or size [15,17–19,25,37]. We did not find any significant changes in the width (right to left) and height of the synchondroses. A possible reason for the observation of unaffected cranial width and height is that the width and height of the growth centers (synchondroses) were not altered by nicotine exposure. Another possible explanation is that because of the growing brain and the non-fusing cranial sutures, the neurocranium growth in height and width was not interrupted and was initiated by the growing brain [52]. To contextualize the magnitude of effect of our results, deficiencies in growth for our significantly altered craniofacial variables span a 5–10% reduction in dimensional growth for this model. As this experiment data only investigated a single timepoint after nicotine exposure we can speculate as to what the consequences are of continued deficiency in craniofacial growth.

The changes we detected in the synchondroses are at least partially driven by the chondrocyte population. Data collected from H&E-stained tissue sections showed significant reduction in the number of chondrocytes of the SOS in both nicotine exposures compared to the controls. Normal growth of the cranial base synchondroses by endochondral ossification takes place through hyperplasia and requires sequential changes of the chondrocytes in the central reserve (resting) zone to proliferate and divide into stacks of chondrocytes in the proliferative zone. Then, these cells enlarge and become hypertrophic and eventually undergo apoptosis as ossification occurs and osteoblasts take over [48,55–57]. In humans, individual synchondroses fuse normally at various ages and their premature fusion is associated with craniosynostosis, skeletal class III facial phenotype, and many other syndromic midface hypoplasias [39,53,58,59]. In mice, synchondroses do not fuse until later in adult life, and significant reduction of chondrocyte populations in the SOS could have a premature fusion – like effect, essentially causing a stasis or cessation in growth. The lactation exposure did not affect the chondrocyte numbers of the ISS, but the pregnancy and lactation exposed mice showed significant reduction of chondrocytes compared to controls. This difference in how SOS and ISS synchondroses respond to maternal nicotine exposure could be explained by the different embryological origins from mesoderm or neural crest cells [57] which is a direction for a future study.

The collagen content of the SOS was significantly altered as a result of nicotine exposure during lactation, but we did not detect any significant changes in the collagen content of the ISS. This may be a logical result of the effects of nicotine on the form and composition of the independent synchondroses. Our lab previously published data [10] showing nicotinic Acetyl choline (Ach) receptors in the synchondroses and others have found cell specific effects to nicotine exposure including to cartilage cells [17–19,25,36,37].Future experiments should investigate the possibility of nicotine binding to Ach receptors on cells in the synchondroses and interfering with proliferation or altering chondrocytes’ ability to produce collagen. It has also been shown that alterations of collagen binding to chondrocytes by knocking out DDR2 (discoidin domain receptor 2) which is a cell receptor for collagen caused deficient anteroposterior length of the skull and cranial base of mice [60]. Although our data did not show how collagen content was reduced with nicotine exposure, we now know that healthy collagen – chondrocytes interaction is vital for chondrocyte function in synchondroses as the growth centers driving anteroposterior craniofacial growth. In the SOS, we found significantly less intermediate and more mature collagen fibers with lactation exposure compared to the controls, but no significant differences for the ISS. These results show specificity of alterations in SOS compared to ISS which could be caused by the different embryological origin or point out a more active role of SOS in driving postnatal growth in mice [57]. In humans, the SOS is responsible for the majority of postnatal growth at the cranial base [61]. This may explain the disparity in changes observed here between the two synchondroses in response to nicotine exposure. Nonetheless, our work and that of others make it plain that the effects of nicotine are multi-factorial, far-reaching, and potentially long lasting [8,10,13–21,25,35–37].

Overall, it is clear from our findings as well as the literature, that maternal nicotine exposure alters craniofacial growth and is potentially harmful to offspring [8–10,13–18,20,25,35–37]. Specifically, alterations in the anteroposterior growth trajectory, and the form and composition of SOS indicate that postnatal exposure to nicotine via breastmilk is enough to potentially alter craniofacial form. It is still not clear why the lactation period appears to be more critical for exposure to maternal nicotine compared to the longer period of pregnancy and lactation, but a possible explanation is that developing tissues adapt to nicotine exposure in the intrauterine environment and will not be as detrimentally affected as an acute exposure after birth which is a mechanism we will interrogate in our future experiments. Additionally, the mechanism of action for the effects of nicotine, such as inhibition of potassium channels may be cell type and developmental timing specific [17,18,25,35–37,62,63]. Regardless of mechanism, these data add to the growing body of literature indicating that nicotine exposure such as that facilitated by ENDS is not in fact safer than traditional smoking as there is a potential for significant and lasting effects of any nicotine exposure.

## Supporting information

S1 Table Sex as an Independent Variable. Sex was considered as an independent variable for each growth measure studied. Data was screened for normality and homogeneity of variance. If assumptions were met, a Two-Way Test was used to determine if there were significant difference by sex or if there was a significant interaction term for sex by exposure for each growth variable. If normality was violated a Friedman’s test was carried out in a similar fashion using ranked data for those variables. For all growth variables studied, there were no significant differences by sex. There were no significant interaction terms for sex by exposure indicating exposure effected both sexes similarly. These data suggest no segregation by biological sex for response to nicotine exposure.
(DOCX)

S2 Table Litter as a Covariate. Litter was considered as covariate for the cephalometric analyses performed for each growth measure studied. Data was screened for normality and homogeneity of variance. If assumptions were met, an ANCOVA was used to determine if there was influence of litter as a covariate. If normality was violated, non parametric ranked adjusted data was assessed for those variables. For all growth variables studied, ANCOVA reveled litter was not significant with the exception of for the anterior facial width. To assess Anterior Facial Width in the context of litter as a significant covariate the same relationships observed in Figure 1 were confirmed using QUADE nonparametric Analysis of Covariance, F = 5.750, p = 0.005, with post-hoc pairwise comparisons confirming pregnancy and lactation exposure to be significantly wider than control (p = 0.037) or lactation only (p = 0.001) respectively.
(DOCX)
