# Supplementary material for: Exposure to maternal nicotine in utero and/or via lactation alters craniofacial development in mice
Source: PLoS One. 2025 Aug 1;20(8):e0329403. doi: 10.1371/journal.pone.0329403 (PMC12316278; doi:10.1371/journal.pone.0329403)
Supplement: S1 Table — Sex was considered as an independent variable for each growth measure studied. Data was screened for normality and homogeneity of variance. If assumptions were met, a Two-Way Test was used to determine if there were significant difference by sex or if there was a significant interaction term for sex by exposure for each growth variable. If normality was violated a Friedman’s test was carried out in a similar fashion using ranked data for those variables. For all growth variables studied, there were no significant differences by sex. There were no significant interaction terms for sex by exposure indicating exposure effected both sexes similarly. These data suggest no segregation by biological sex for response to nicotine exposure. (DOCX) [file pone.0329403.s001.docx]

**Supplemental Table 1: Sex as an Independent Variable.**

| **Growth Variables** | **Sex as an Independent Variable** | **Sex x Growth Variable Interaction Term** |
| --- | --- | --- |
| Weight | F=0.002, p=0.965 | F=1.826, p=0.169 |
| Cranial Length | F=0.623, p=0.433 | F=0.507, p=0.605 |
| Craniofacial Length | F=0.009, p=0.924 | F=1.162, p=0.320 |
| Cranial Height | F=2.020, p=0.160 | F=2.793, p=0.069 |
| Cranial Width | F=3.092, p=0.083 | F=0.996, p=0.375 |
| Anterior Facial Width | F=1.824, p=0.181 | F=2.141, p=0.126 |
| Mid Facial Width | F=1.204 p=0.277 | F=0.241, p=0.787 |
| Posterior Facial Width | F=0.001, p=0.978 | F=1.696, p=0.191 |
| Cranial Base Length | F=0.157, p=0.694 | F=2.795, p=0.073 |

Sex was considered as an independent variable for each growth measure studied. Data was screened for normality and homogeneity of variance. If assumptions were met, a Two-Way Test was used to determine if there were significant difference by sex or if there was a significant interaction term for sex by exposure for each growth variable. If normality was violated a Friedman’s test was carried out in a similar fashion using ranked data for those variables. For all growth variables studied, there were no significant differences by sex. There were no significant interaction terms for sex by exposure indicating exposure effected both sexes similarly. These data suggest no segregation by biological sex for response to nicotine exposure.
