# Supplementary material for: Exposure to maternal nicotine in utero and/or via lactation alters craniofacial development in mice
Source: PLoS One. 2025 Aug 1;20(8):e0329403. doi: 10.1371/journal.pone.0329403 (PMC12316278; doi:10.1371/journal.pone.0329403)
Supplement: S2 Table — Litter was considered as covariate for the cephalometric analyses performed for each growth measure studied. Data was screened for normality and homogeneity of variance. If assumptions were met, an ANCOVA was used to determine if there was influence of litter as a covariate. If normality was violated, non parametric ranked adjusted data was assessed for those variables. For all growth variables studied, ANCOVA reveled litter was not significant with the exception of for the anterior facial width. To assess Anterior Facial Width in the context of litter as a significant covariate the same relationships observed in Figure 1 were confirmed using QUADE nonparametric Analysis of Covariance, F = 5.750, p = 0.005, with post-hoc pairwise comparisons confirming pregnancy and lactation exposure to be significantly wider than control (p = 0.037) or lactation only (p = 0.001) respectively. (DOCX) [file pone.0329403.s002.docx]

**Supplemental Table 2: Litter as a Covariate.**

| **Growth Variables** | **Litter as a Covariate** |
| --- | --- |
| **Weight** | **F=3.811, p=0.055** |
| **Cranial Length** | **F=0.003, p=0.955** |
| **Craniofacial Length** | **F=0.392, p=0.533** |
| **Cranial Height** | **F=2.734, p=0.103** |
| **Cranial Width** | **F=0.354, p=0.554** |
| **Anterior Facial Width** | **F=10.312, p=0.002**** |
| **Mid Facial Width** | **F=1.527 p=0.221** |
| **Posterior Facial Width** | **F=0.422, p=0.518** |
| **Cranial Base Length** | **F=0.283, p=0.598** |

Litter was considered as covariate for the cephalometric analyses performed for each growth measure studied. Data was screened for normality and homogeneity of variance. If assumptions were met, an ANCOVA was used to determine if there was influence of litter as a covariate. If normality was violated, non parametric ranked adjusted data was assessed for those variables. For all growth variables studied, ANCOVA reveled litter was not significant with the exception of for the anterior facial width. To assess Anterior Facial Width in the context of litter as a significant covariate the same relationships observed in Figure 1 were confirmed using QUADE nonparametric Analysis of Covariance, F=5.750, p=0.005, with post-hoc pairwise comparisons confirming pregnancy and lactation exposure to be significantly wider than control (p=0.037) or lactation only (p=0.001) respectively.
